# A magpie with a card-index mind – Charles Davies Sherborn 1861–1942

**DOI:** 10.3897/zookeys.550.9975

**Published:** 2016-01-07

**Authors:** Karolyn Shindler

**Affiliations:** 1 Library Associate, Natural History Museum, London, United Kingdom

## Abstract

Charles Davies Sherborn was geologist, indexer and bibliographer extraordinaire. He was fascinated by science from an early age and like so many Victorians, the young Sherborn was a passionate natural history collector and was obsessed with expanding his collection of land and freshwater shells. He later described himself as being a ‘thorough magpie’ and having ‘a card-index mind’, and these two traits coalesced in his monumental *Index Animalium*, the compilation of which occupied 43 years of his life. One of the first visitors through the doors of the Natural History Museum in South Kensington when it opened in 1881, Sherborn began work there seven years later as one of the small band of unofficial scientific workers, paid by the number of fossils he prepared. By the time of his death in 1942, Sherborn’s corner in the Museum was the first port of call for generations of scientists seeking advice, information – or an invitation to one of his famous ‘smoke and chat’ parties.

In addition to his work on the *Index*, Sherborn is also responsible for rescuing from damp and probable destruction the huge archive of Sir Richard Owen, the great comparative anatomist and the prime mover behind the creation of the Natural History Museum, London. Without Sherborn, this invaluable resource of correspondence, manuscripts and books may well have been irretrievably ruined.

Charles Davies Sherborn’s fascination with science began early. Like many small boys he collected rocks and fossils and was obsessed by expanding his collection of land and freshwater shells. Few boys, however have attempted to construct volcanoes in their gardens in west London, the consequent explosion resulting in a visit from the police. Sherborn’s life was never going to be ordinary – even his first flat when he left home was above an undertaker’s, with wood chippings from the coffins the fuel for his fire.

But it was his passion for collecting that triumphed – his ‘magpie habits’ as he called it – and was to result in his spectacular work of bibliography, the *Index Animalium*, a true labour of love (and shamefully little financial reward). His object was to provide zoologists with a complete list of all the generic and specific names that had been applied to animals from 1 January 1758, giving a reference to the book or journal in which it was first published, and the date of publication. It was to occupy 43 years of his life.

Two years after he had embarked on the *Index*, Sherborn received an extraordinary and challenging invitation. He was asked by the Reverend Richard Startin Owen to sort through and organise the papers of his grandfather, the great anatomist and creator of the Natural History Museum, Professor Richard Owen, who died in 1892. The archive was vast – and in a terrible state, heaped up, vulnerable to damp and rats and in urgent need of rescue. It was a massive task, involving tens of thousands of letters, manuscripts and books.

Simply one of these undertakings would have defeated most people, but for this self-educated, extraordinarily generous man with an encyclopedic brain, this was very heaven.

Charles Davies Sherborn was born in Chelsea on 30 June 1861, the eldest child of Charles William Sherborn, a renowned etcher and line-engraver, and his wife, Hannah. Sherborn was sent to school at the age of three, but his formal education ended abruptly when he was 14: his father’s business misfortunes meant he had to leave school and earn a living. But he was already, like so many Victorians of all ages, a passionate natural history collector, particularly of rocks and fossils and was obsessed with expanding his collection of land and freshwater shells.

His first job was in an upmarket stationery and bookshop in Bond Street, which Sherborn later claimed laid the foundation for his expertise in bibliography (Cleevely 2004). He did not, though, give up on science. His next job was as a clerk in a tailor’s near the Museum of Practical Geology in Jermyn Street in London.

His spare time was spent there, or reading in the library of the Victoria and Albert Museum – which he always called by its old name, the South Kensington Museum – while his weekends would be occupied in fieldwork. When the Natural History Museum opened at Easter 1881, Sherborn maintained he was one of the first half dozen visitors through its doors.

Two years later, he met the retired Professor of Geology at the Royal Military College, Sandhurst, Thomas Rupert Jones, who asked Sherborn for help with papers he was writing on microscopic fossils known as foraminifera (Miller 2016). By 1887 – all in his spare time – they had published three papers together, with Sherborn, who had considerable artistic talent, doing the drawings.

**Figure 1.**
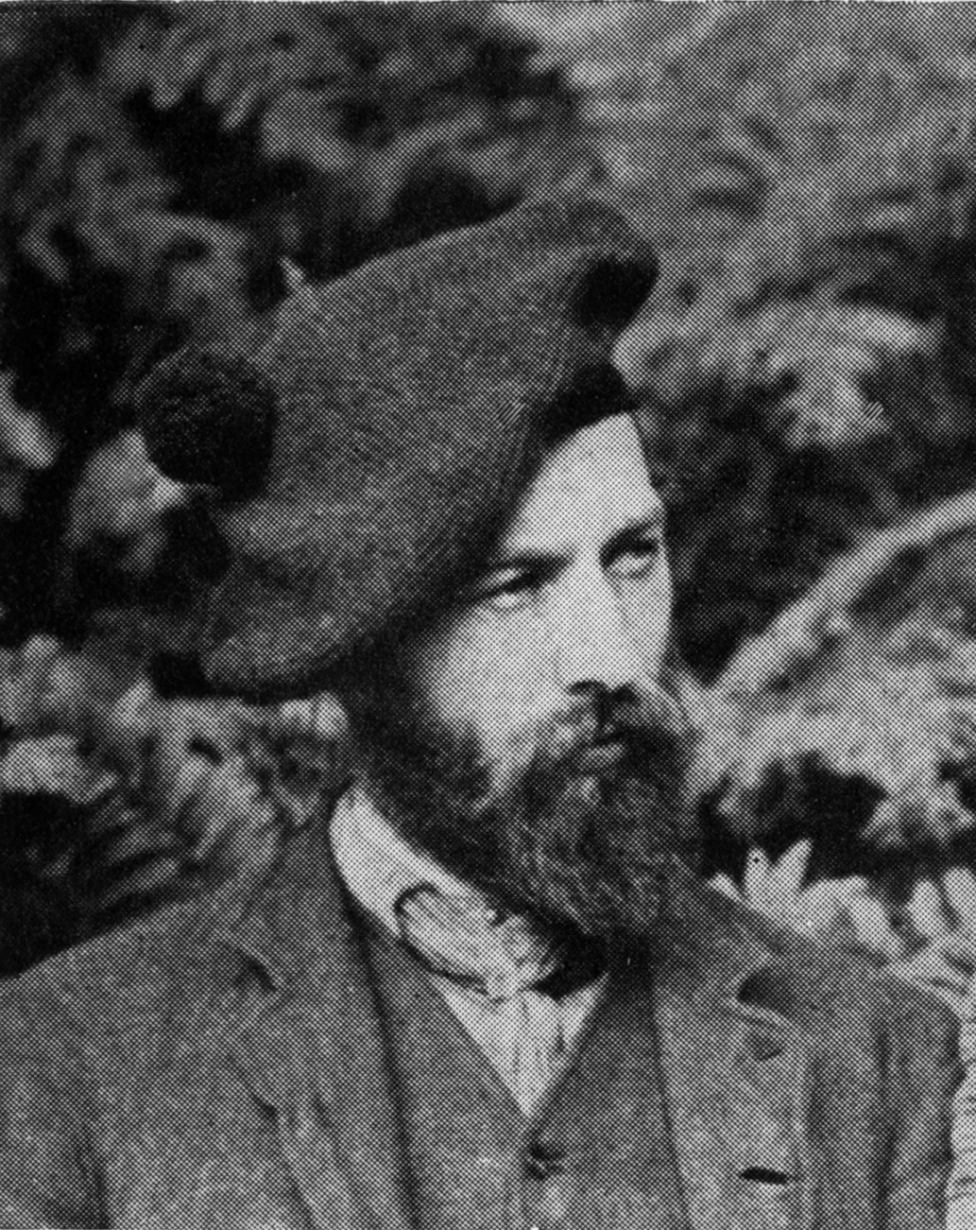
Sherborn at the age of about 25. This, according to his friend and biographer JR Norman, encapsulates ‘Charles in the Geological Excursion Days’. (With permission of The Trustees of the Natural History Museum, London)

**Figure 2.**
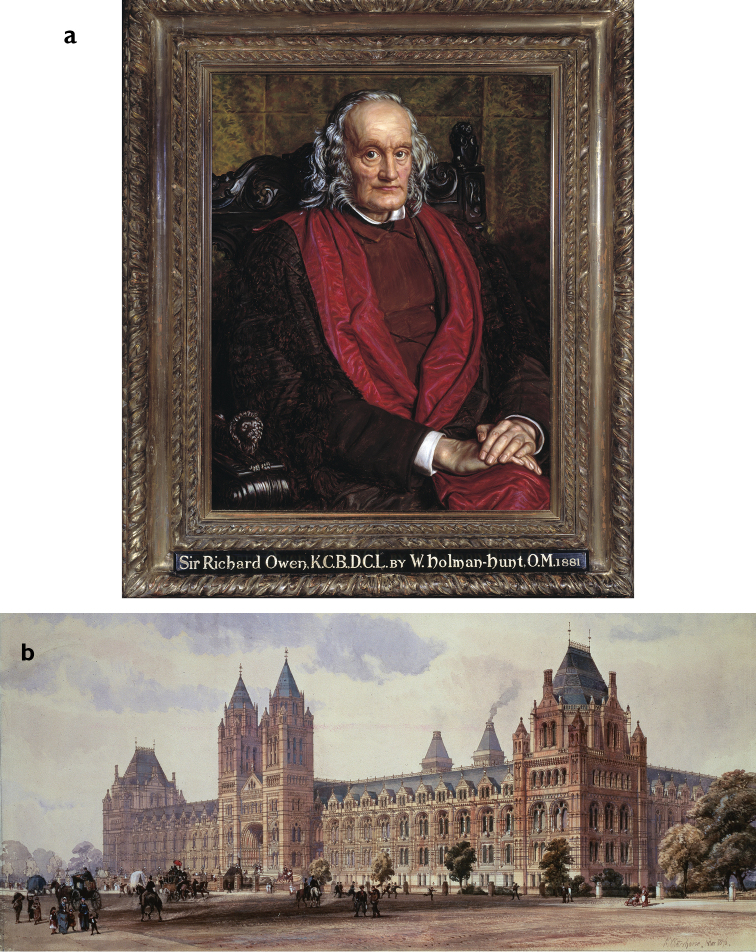
**a** Sir Richard Owen (1804–1892) in a portrait of 1881 by the Pre-Raphaelite painter William Holman Hunt (1827–1910). Owen’s vision of a museum for natural history was realised that year with the opening of **b** the British Museum (Natural History) in South Kensington, which immediately became known as the Natural History Museum. This original illustration is by the Museum’s architect, Alfred Waterhouse (1830–1905). During construction, the Treasury objected to the cost, and Waterhouse suggested it should be built in two stages, first the magnificent front, then later the back and two wings - one along Exhibition Road that you can just see in the drawing, and along Queen’s Gate. With the front completed, the will to fund stage two vanished and the wings was never built. (With permission of The Trustees of the Natural History Museum, London)

**Figure 3.**
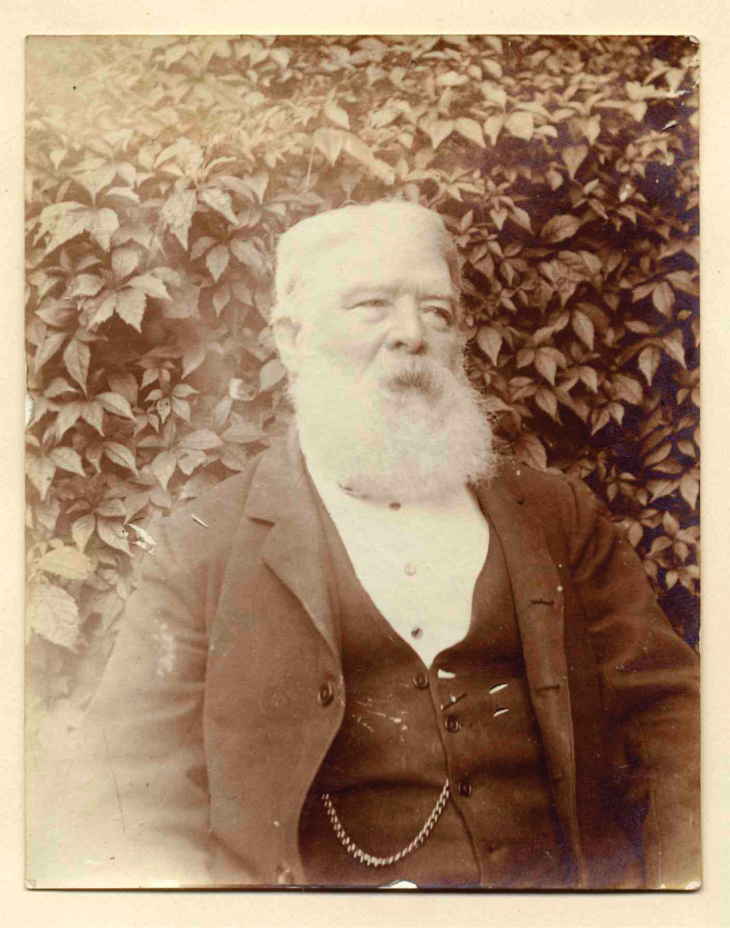
The geologist and palaeontologist Thomas Rupert Jones (1819–1911) was Sherborn’s scientific mentor and colleague. In 1864 he founded the *Geological Magazine* with Dr Henry Woodward of the British Museum’s Geological Department.

Prompted by the great number of journals they had had to consult, with Rupert Jones’s encouragement Sherborn began to compile *A Bibliography of the Foraminifera* which was ready for publication in 1888. An American work on the same subject was published at this time, which Sherborn felt was so poor that he wrote a vitriolic criticism of it which was published in the journal *Nature* under the heading, 'An “Instructive” Bibliography of the Foraminifera'. According to Sherborn, it was 'absolutely untrustworthy', 'comparatively useless', with 'serious defects for which excuse must be difficult'. Furthermore, 'many of these errors and defects might have been avoided', he ended witheringly, 'had the compiler been used to public libraries' (Sherborn 1888). However justified Sherborn's criticism, the consequence for him was potentially disastrous. When he applied to the Royal Society for a grant for £100 for printing costs for his own *Bibliography*, it was refused on the grounds that he had written ‘a savage criticism of a foreigner’. It was only the generous response of a publishing friend of his that ensured his work was published and, as Sherborn later wrote, ‘wiped out completely the churlish action of the Royal Society’.

In 1888 Sherborn began part-time work in the Geology department at the Natural History Museum, preparing and cleaning fossils. He was paid according to the number of fossils he worked on. Quite literally, that changed his life. He loved the work, loved being in the world of natural history and, financially precarious though this was, he gave up his full-time employment – by this time he was working as a secretary at the Middlesex Hospital – and determined to try to earn a living through science. He became an unofficial scientific worker at the Museum, paid according to the amount of work he did. He never joined the staff.

**Figure 4.**
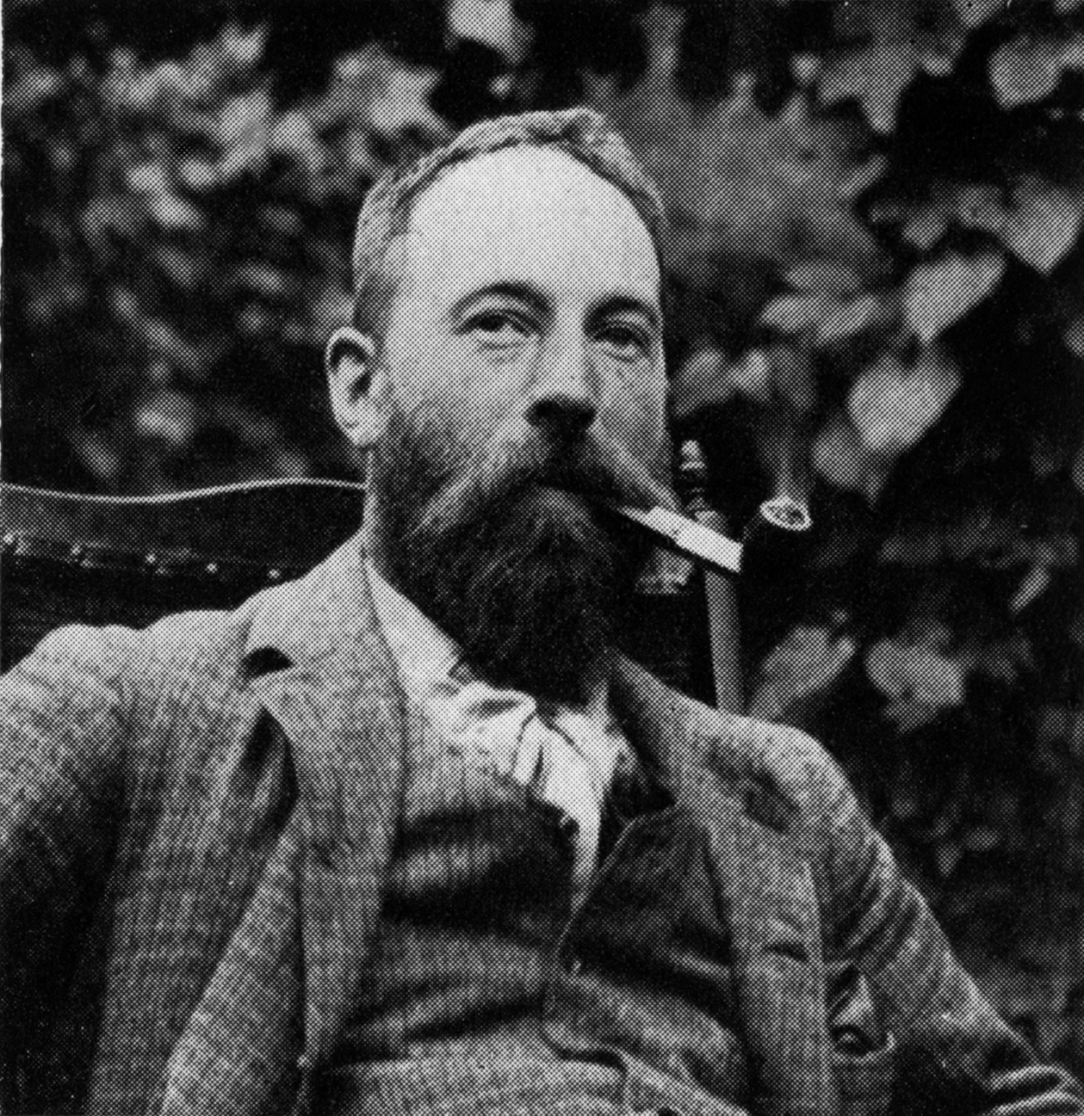
Sherborn aged 32. He was rarely seen without his beloved pipe.: (With permission of The Trustees of the Natural History Museum, London)

His passion at first was geology and palaeontology, but through his work with Rupert Jones, and then his collaboration with the palaeontologist Arthur Smith Woodward at the Natural History Museum, he found himself increasingly drawn to scientific bibliography. There was, he discovered, an overwhelming need for zoologists and palaeontologists to have available to them a complete index of all scientific names applied to animals, living and extinct, giving the exact date and place of publication. In the mid-18^th^ century, the Swedish naturalist, Carl Linnaeus brought order to the chaos of natural history names with his binominal (often called binomial) solution: giving everything living – plants and animals - two Latin names, the genus or generic name, and the trivial or specific name – a descriptive one. Inspired by his own *Bibliography of the Foraminifera* and a work endowed by Charles Darwin, the *Index Kewensis*, which was devoted to plants, Sherborn put forward a plan for an *Index Animalium*. The 10^th^ edition of Linnaeus’s work, published in 1758 was regarded as definitive and that is why Sherborn begins his monumental work then. His original plan was to have ended the *Index* in 1899. He took advice from many scientists, and wrote to *Nature* to announce the project and that he would be starting work on 1 July, 1890 – which he duly did (Sherborn 1890). Endearingly, he noted later that his friends considered his rigorous, precise work ‘incredibly dull’, although ‘it gave me a lot of pleasure’ (see Evenhuis (2016), on ‘the Indexer’s Mind’).

No one had ever attempted anything on this scale before, and, as he noted in 1896, ‘The vastness of the record is appalling’. He went on to remark with unusual optimism that, ‘given time all difficulties disappear’, although whether he would have agreed with that sentiment 30 years later is another matter (Sherborn 1896). His first task was to work out how to tackle it. In an exercise book with a shiny reddish-brown cover, now a bit tattered, Sherborn painstakingly evolved his own rules. He noted problems as they arose – ‘Question of Double-barrelled names!!’ – and methodically worked out through the pages ways they might be resolved. ‘How far’, he asked himself, ‘can one accept authors who use one, two or three words as a specific term?’ The issue of sub genus occupies a number of pages and clearly caused him considerable trouble, ‘it is absolutely impossible’, he fumed, ‘to get unanimity among authors, & often the author has not the dimmest idea what he himself means!’ (Sherborn Collection nd)

When he embarked on this project, he was also working on labelling and registering type specimens in the Geological Society and preparing fossils at the NHM during the day – both of which gave him an income. The index he worked on only at night, taking the books he was working on home to his flat.

Yet in one year, he had worked through every page of some 500 volumes and indexed 40,000 names. ‘References’, he wrote, ‘are taken from one book at a time – i.e., a book is gone through from cover to cover – every genus and species, and every change of genus, being systematically recorded; thus completely disposing of that particular book, and ensuring the almost absolute certainty of every reference being taken’. (Sherborn 1896).

**Figure 5.**
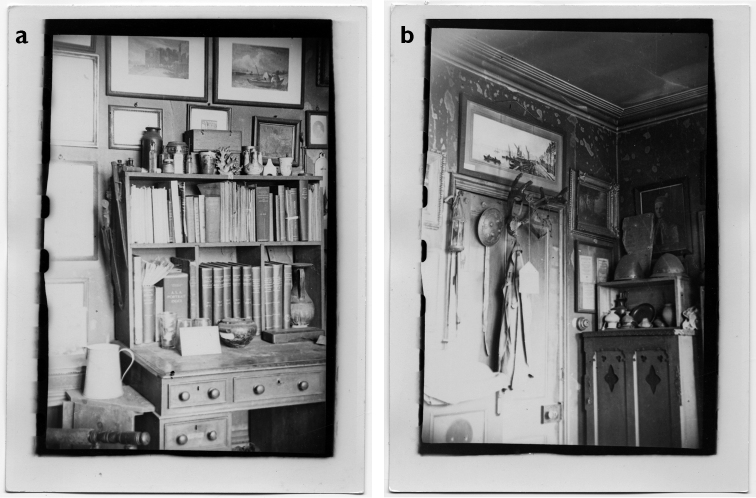
Sherborn’s house at 49 Peterborough Road, London SW6 was a wonderful magpie’s nest, filled with his eclectic and wide-ranging collections. These photos were probably taken after his death in 1942. (With permission of The Trustees of the Natural History Museum, London)

**Figure 6.**
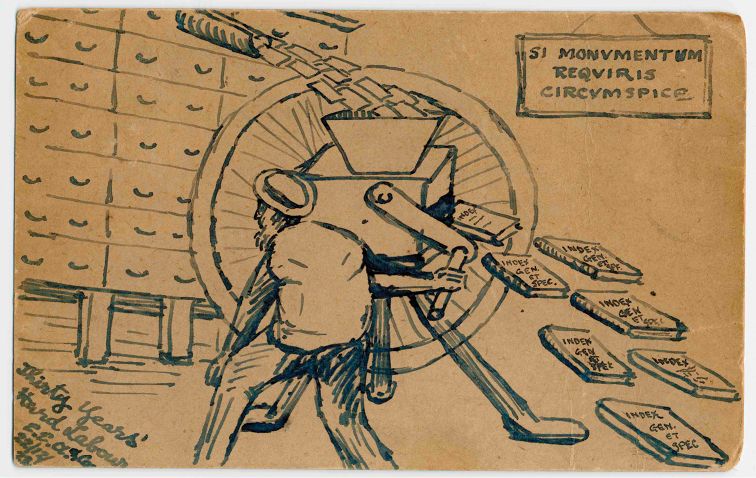
This splendid cartoon is of CDS operating a machine which turns his index cards into books. Hanging on the wall is the famous Latin inscription from Sir Christopher Wren’s tomb in St Paul’s Cathedral (which of course he designed): ‘*Si monumentum requiris circumspice*’ - If you seek his monument, look around you. In the bottom left corner is ‘Thirty Years Hard Labour, E.E.A.’. The cartoon is a postcard on which is written on the other side, ‘Best of Good wishes for Christmas and the New Year, from all at Evaeria. 22.xii.23’. This is the name of the house of Major Ernest Edward Austen, DSO, FZS(1867–1938), assistant keeper of the NHM’s Entomology Department and an authority on tsetse flies. (With permission of The Trustees of the Natural History Museum, London)

The statistics of this work are staggering. The 11 volumes, totalling more than 9000 pages, contain about 440,000 names, extracted by Sherborn from thousands of academic books and journals, in many different languages, each naming newly discovered species.

Each name, with the book or journal in which it was first published and the date, he recorded in black lead pencil on a small slip of paper, 127mm by 63mm (5 inches by 2^1/2^ inches) and then duplicated. Carbon-paper – blue or green – was soon used to make the duplicates, ‘both methods having proved to be quite indelible’, he wrote (Sherborn 1896). By 1916 there were more than one million slips. It took him one month to edit 10,000 and one hour to number 1500 of them.

From the outset, the *Index Animalium* project was hampered by lack of funds. Although the NHM gave him his own space in the museum’s library, he was reliant on grants from scientific institutions. These were barely of subsistence level. In 1892, the British Association for the Advancement of Science which to start with provided most of the funds, set up a committee to protect Sherborn from the added burden of administering the money. Year after year the committee wrote to various bodies pleading for funds so Sherborn could employ ‘even a boy to do the sorting, alphabetical arrangement and numbering of slips’, but the extra money never came (BAAS 1896-1912). Everything had to be done by Sherborn alone. It took three years just to put the million or so slips into rough alphabetical order.

**Figure 7.**
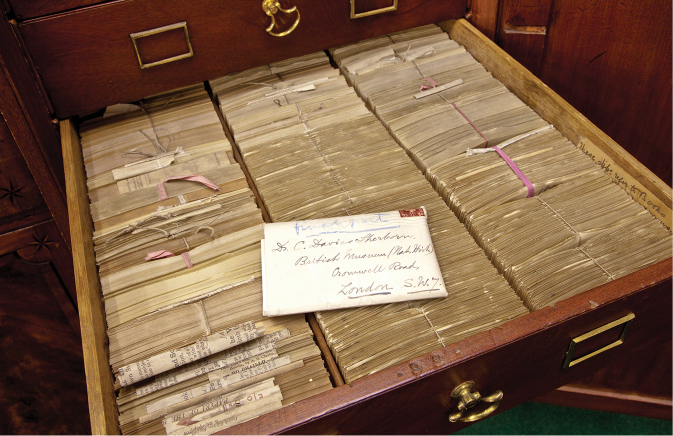
This is just one of the drawers of Sherborn’s index cards. It is now preserved in the NHM’s Library. Sherborn recorded in pencil each name, with the book or journal in which it was first published and the date, on a small slip of paper, 127mm by 63mm, and then duplicated it. It took him one month to edit 10,000 and one hour to number 1,500 of them. By 1916 there were more than one million slips. It took 3 years to put them into rough alphabetical order. These cards were for the years 1850–1899, and were never published. The volume of material for those years was so great it would have demanded a team of workers to complete the task, not solely CDS, and that was simply unaffordable. (With permission of The Trustees of the Natural History Museum, London)

**Figure 8.**
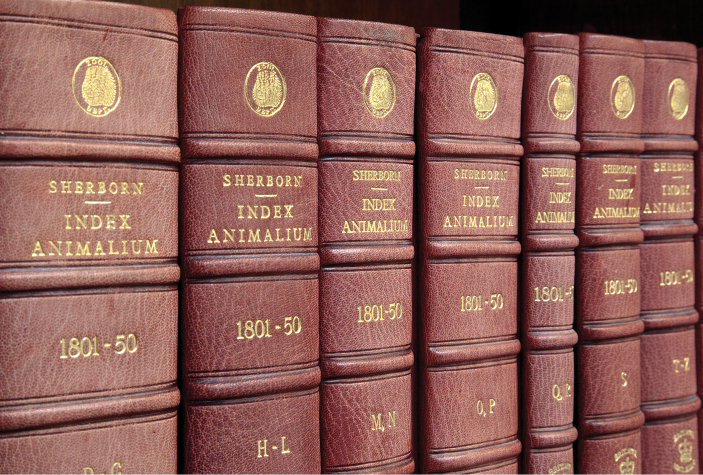
Some of the 11 volumes of Sherborn’s *Index Animalium*, held in the NHM’s Library. They total more than 9000 pages, and contain about 440,000 names. (With permission of The Trustees of the Natural History Museum, London)

**Figure 9.**
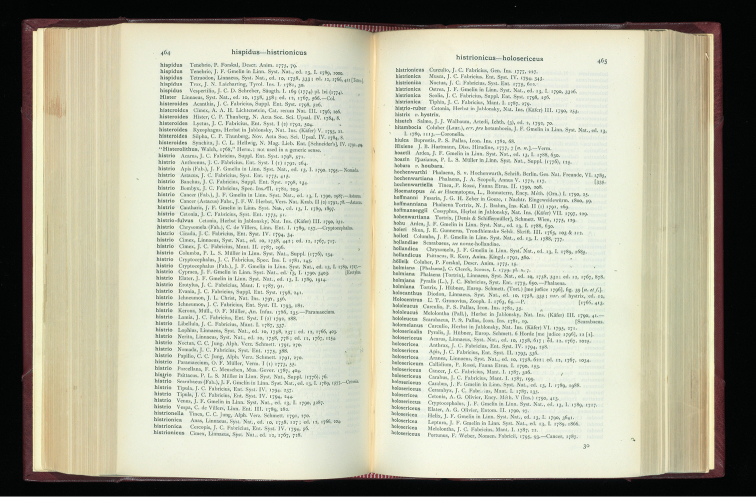
Pages from the first part of *Index Animalium*, 1758–1800. (With permission of The Trustees of the Natural History Museum, London)

In all those 43 years of labour, Sherborn received a total of just £5415 – or an average of £126 a year – in grants. It was not until 1912 that the Natural History Museum finally assumed responsibility for Sherborn – something for which his friends had been angling for some time. In 1909 the Keeper of Zoology, Sidney Harmer, raised the question with colleagues, but was discouraged by the museum's Secretary, Charles Edward Fagan, who thought the museum's finances were such that 'the moment is not a propitious one' (Harmer 1909). It was to be another three years before the Trustees awarded him an annual grant. In 1912 it was £100 a year. By 1931 that had risen to £250, where it remained until his death.

The first volume of the *Index* was published in 1902 – it took about 20 months to print the 1200 pages – and covers the years 1758–1800. Although twelve years had elapsed since he started work, the first volume was the easy bit, though he did not know it at the time, as he wrote in December 1902 to a friend, Ernest Hartert – a curator at Walther Rothschild’s museum at Tring: ‘I am so glad it is out, it has cost me much work & ill health, but I feel like a giant refreshed now I have something to SHOW’. (Sherborn 1902b).

**Figure 10.**
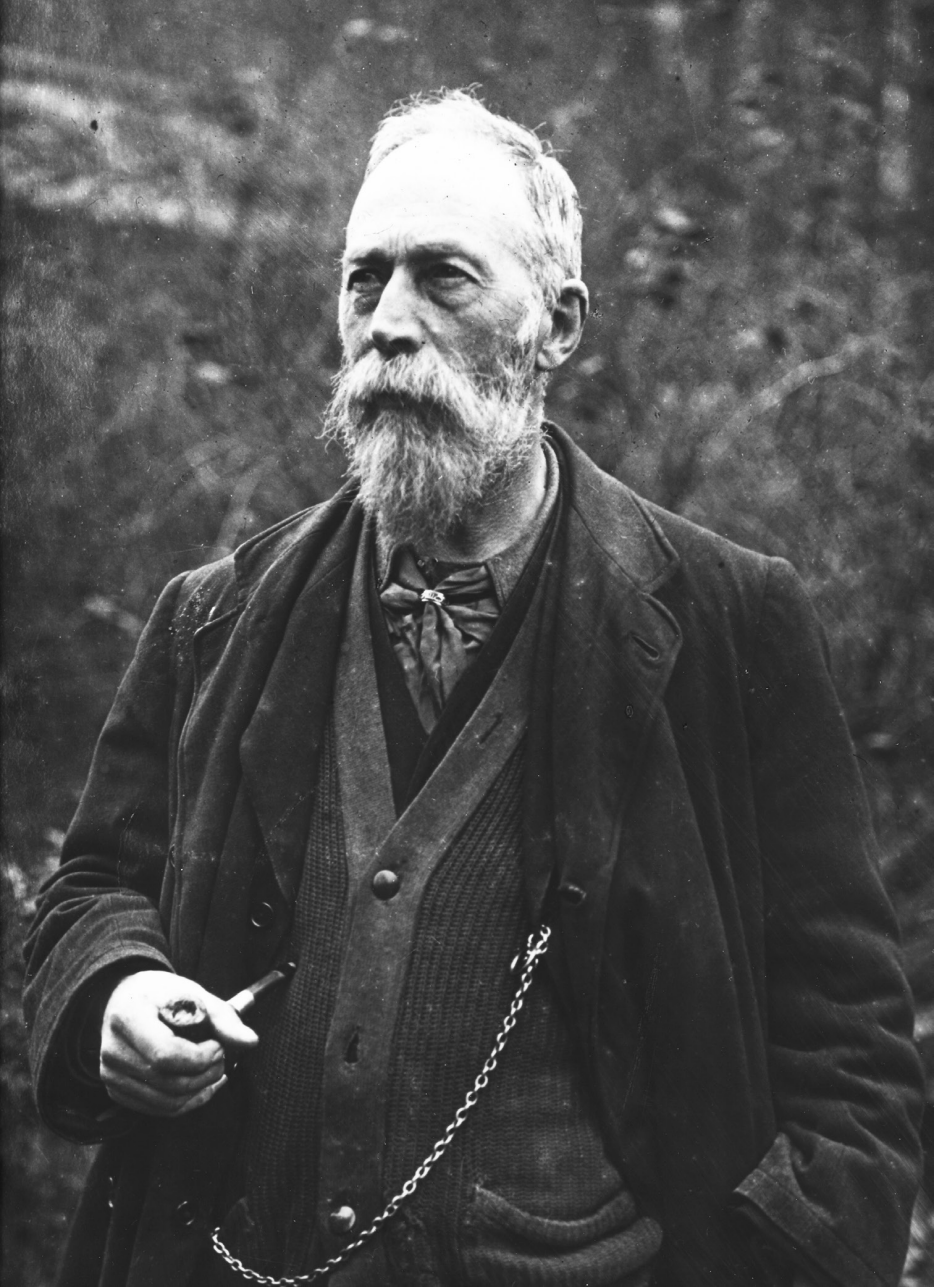
Sherborn at the age of 61. He was frugal and cared little for his appearance. He felt the cold keenly, and would keep warm by inserting a sort of apron of felt beneath his many layers of clothes. In extremis, he would also use a newspaper. Instead of a necktie, he wore a piece of folded red or black material, held together with an old gold ring. The black material, his biographer JR Norman discovered, was cut from an old umbrella. (With permission of The Trustees of the Natural History Museum, London)

So severe was his illness, however, that he refers to it in the introduction to the first volume, recording that, ‘an unfortunate breakdown in health, which has frequently recurred, laid me aside for three years, and thus the actual time spent on the manuscript has amounted to eight years” (Sherborn 1902). He was left with raging headaches and eye problems.

His ill health, however, was only partly due to his labours on the *Index*. Just two years after he began work on it, he received an extraordinary invitation. He was asked to collaborate on writing the biography of the great comparative anatomist, creator of the Natural History Museum and its first Superintendent, Sir Richard Owen. The invitation was from Owen’s grandson, the Reverend Richard Startin Owen.

Sherborn had first met Sir Richard Owen in October 1889, when he was invited to dine at his home in Sheen Lodge, Richmond Park. Owen was then 85 and in poor health. As a memento of the occasion, Sherborn kept a photograph of Owen, inscribed on the back ‘a memory of an interesting occasion dined with Richard Owen, Oct 27^th^ 1889. C. Davies Sherborn.’ (Sherborn 1887–1942).

If somewhere Sherborn wrote more than that, sadly it does not appear to have survived. Presumably at the dinner he would have met Owen’s grandson, the Reverend R S Owen, and he may have been invited into Owen’s over-flowing, book-lined study. He may even have been given a preliminary glimpse of the 60 years worth of manuscripts, correspondence and books that lay neglected and unsorted.

Sir Richard Owen’s career had begun in 1827 when he became assistant conservator at the Hunterian Museum at the Royal College of Surgeons. After his retirement 56 years later, as Superintendent of the new Natural History Museum, the contents of his office – including two cartloads of papers – followed him home to his grace-and-favour residence, Sheen Lodge. This mass of personal and professional correspondence, manuscripts and books led Owen to exclaim, ‘I am compelled to part with a Gardener and to turn his cottage into their receiving house’.

**Figure 11.**
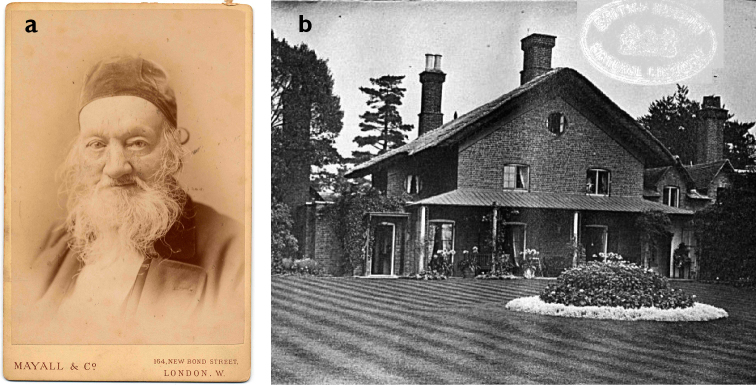
**a** Sir Richard Owen gave this photograph to Sherborn. CDS has written on the reverse: ‘a memory of an interesting occasion dined with Richard Owen, Oct 27^th^ 1889 C. Davies Sherborn’. The dinner took place at **b** Sheen Lodge, Sir Richard’s grace-and-favour home in Richmond Park. (With permission of The Trustees of the Natural History Museum, London)

What was to become of it all became an increasingly pressing problem as Owen’s health declined. His beloved wife Caroline was dead, his only son William had apparently committed suicide in 1886, and none of his seven grandchildren, who lived with him at Sheen Lodge, appeared to have any interest in science, nor had they any idea of what in this huge mass of paper might be of importance or indeed, value. Unlike Charles Darwin’s friends and family who brilliantly preserved and archived his papers, understanding full well Darwin’s importance, there was no one with the inclination or knowledge to do this for Owen.

It was at some point after the dinner in 1889, that Sherborn was asked by Reverend RS (Richard Startin) Owen to sort through his grandfather’s books and organise their sale. As Sherborn commented in his own autobiographical notes, Owen – or the family – had already sold a good number, ‘so there remained only some ten years accumulation’. Sherborn also spent many hours with Owen (Norman 1944). Since his son William’s death in 1886, Owen had become increasingly withdrawn. His beard grew, his hair, as photographs show, was straggly and unkempt. His ‘great glittering eyes’ that Thomas Carlyle had remarked upon, were huge in his gaunt face. His appearance frightened his younger grandchildren, but to Sherborn he reminisced and gossiped about ‘all the great men of his youth’.

It was possibly not that surprising then, that in August 1892 – when Owen was so ill he could not speak or swallow – Sherborn received the invitation which, to say the least, clearly thrilled him. On 20 August 1892, he wrote to the librarian of the Natural History Museum, Bernard Barham Woodward, ‘Some men are born great... and some have greatness thrust upon them... and I therefore entrust to you as a special friend the fact that I was summoned on Tuesday morning to East Sheen and asked to collaborate with the Rev R O on the Life and Letters of Richard Owen’. You can almost hear the glee in his voice. He urged Woodward to be discreet with the news. ‘All is yet a secret, as by no means must it get to the Press until matters are settled.’ There is no correspondence to show how well Sherborn had come to know the Owens, but he added, ‘I am very proud that I should have been chosen by the family after all these years; and not only chosen but thanked again and again for all my kindness when first I went down there’. Spending hours talking to the frail professor was presumably what they had in mind, but as Sherborn noted, his conversations with Owen would be of ‘great value’ for the biography (Norman 1944).

When he wrote to BB Woodward, Sherborn was already hard at work sorting Owen’s papers, and had nearly disentangled the correspondence from the manuscripts. He had, so far, found around 10,000 letters. Given the scientific and historical importance of the material, the condition in which the papers were kept is staggering. Sherborn found the papers in urgent need of preserving and sorting. They were ‘in a cow-shed, exposed to rats and rain’, and this was no exaggeration (Owen Collection, OC62 General Library, NHM). The manuscripts were piled four metres high, while the correspondence filled many packing cases. But for Sherborn, who described himself as having a card-index mind, this was very heaven.

**Figure 12.**
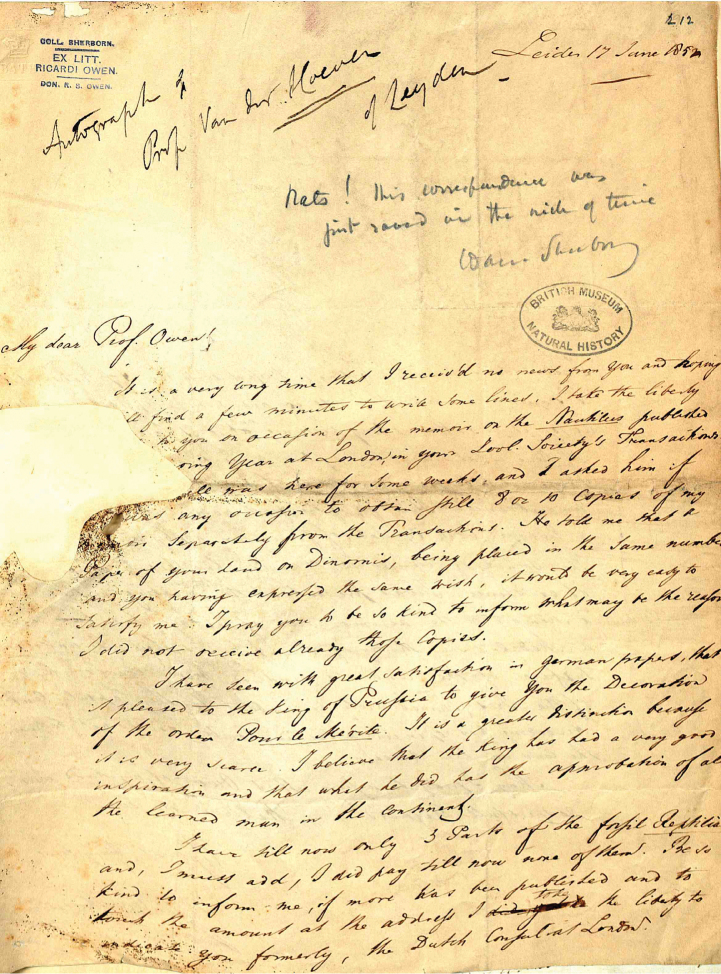
One of the many letters from the Owen correspondence that Sherborn found in urgent need of preserving and sorting. On it he has written ‘Rats! This correspondence was just saved in the nick of time’. (With permission of The Trustees of the Natural History Museum, London)

He told BB Woodward that so important was the Owen biography that, ‘I must husband all my time and strength now, for it is a giant’s task set before me, and this must cap, not sink below, my other works’.

Just two weeks after Sherborn’s ecstatic letter to BB Woodward, on 3 September 1892 the Reverend Owen wrote to Sherborn with news that must have wounded him deeply: without further discussion he was withdrawing the invitation to collaborate on the biography. What appears to have changed Richard S Owen’s mind was his consultation with some of his grand-father’s old friends, including Sir Richard Owen’s successor as Director of the Museum, Sir William Flower. They had concluded that as the biography was not to be a narrative of Owen’s scientific life, but rather a record of his private life, ‘the narrative will be drawn principally from the joint diaries kept by Sir R and his wife. I find that these are of so purely a family & private nature as to compel me to do all the work of extracting & compiling myself’, the Reverend Owen told Sherborn. Sherborn was still to have a role, however. The Reverend Owen told him that as far as sorting the correspondence was concerned, there was no man ‘better fitted for it than yourself’, and he also wanted Sherborn to ‘undertake to revise any mention of Sir R’s scientific work so as to preclude the danger of error’. He would be paid and his work would be acknowledged in the preface (Owen RS 1892).

Sherborn agreed. As the scientific papers were not to be used in the biography, he dispersed many of them. The medical papers went to the Royal College of Surgeons and others went to the Geological Society, which had published a number of Owen’s reports. John Marr, society secretary, wrote to Sherborn to thank him for the papers, ‘The Librarian has made a selection of those which we do not possess, & the others are being returned to you, in accordance with your request’.

In 1894, the biography was published. The Reverend Owen in the preface thanked Sherborn ‘for lending me much assistance throughout’, and ‘for carefully examining Sir Richard’s correspondence’ (Owen RS 1894). He then, Sherborn wrote, ‘gave me the lot, and it filled a four-wheeler to take home. The manuscripts’, Sherborn added devastatingly, ‘were distributed to those interested all over the world.’ As Sherborn did not compile a list of these, it is impossible to know now what was irretrievably lost. Three years later however, Sherborn discovered that R S Owen had not in fact given him ‘the lot.’ He had removed a large part of the collection and only gave them to Sherborn in 1897 as he was about to go to New Zealand for six months. ‘Can you come round tomorrow morning,’ he wrote to Sherborn on 6 October, ‘and cart off the letters? ... Would you mind giving them houseroom while I am away, & also the prints of Hunter which I fear are being treated in rather a reckless fashion.’ (Norman 1944)

The accommodation that Sherborn was able to provide for them at such short notice was scarcely less reckless. ‘The cupboard full of letters & papers in my back room, the manuscript of Hunter in the safe, the papers in the cupboard in Smith Woodward’s room at the Museum & the Diplomas of Sir R Owen there are held in trust for the Rev Richard Owen & are to be kept until he asks for them. The scientific letters he has promised to me & I intend them for the nation to be preserved at the Natural History Museum under BB Woodward’s care as the Scientific Correspondence of Richard Owen.’

But even that still did not account for the whole archive. Sherborn himself, late in his life, noted briefly that, ‘about 100 letters kept back by his grandson, who sold them at Maggs [the antiquarian booksellers] in 1916. His own letters to his wife and sister[s] kept by his grandson.’ (Norman 1944) Sequences of correspondence were separated. I have read a letter in the British Library, only to find its reply in the Natural History Museum and the reply to that in the Royal College of Surgeons.

Letters from the famous were of course sold. Charles Kingsley, Alfred, Lord Tennyson, Charles Dickens – all were among Owen’s friends, but just about all their correspondence has vanished. Also missing are letters that refer to Owen’s disagreements with Charles Darwin and Thomas Huxley. It is extraordinary to believe in all that correspondence that it was scarcely referred to, yet there is hardly anything. Compare that with the Darwin correspondence where the Darwinites’ antipathy to Owen flows remorselessly in letter after letter. Apart from passing references, virtually all there is in the Owen correspondence is one letter in which Owen criticises Huxley’s ‘base and mendacious nature’ and on this Sherborn has written, ‘This is the only letter I remember in which Owen severely criticized an opponent’.

The scale of Sherborn’s task in sorting through this material was huge. Pages had become separated and had to be re-united, authors – and signatures – identified. He annotated many of the letters. On one from the Director of the National Portrait Gallery, George Scharf in 1889, to Owen’s daughter-in-law expressing his great satisfaction that he had ‘secured’ a portrait of ‘my dear old friend’ Sir Richard, Sherborn has scrawled scathingly, ‘This is nonsense, he first refused it owing to some silly regulation and was told that if they didn’t want it, it should be offered to the RCS. Then he jumped at it.’ (Scharf 1889) Before the first of 64 letters written to Owen by the Marchioness of Hastings, Sherborn has noted her brief biographical details. On a letter that Owen wrote to his wife Caroline concerning their grace and favour house, Sherborn has written, ‘Regarding house at Kew’ and then, worryingly, ‘keep’.

And this of course was one of Sherborn’s main difficulties. Confronted with so many thousands of letters, Sherborn edited rigorously, deciding what was worth keeping and, regrettably for subsequent researchers, what he deemed was not. When he presented the material to the Museum in 1908, Sherborn outlined to the librarian BB Woodward the parameters he had used for his selection. I have to admit to finding this horribly painful to read: ‘I have been carefully through the collection with a special knowledge of the history of science and of the collections of the British Museum (Natural History) and have destroyed several thousand letters of no value. Richard Owen kept everything and the great bulk of those destroyed were letters from tradesmen and similar unimportant persons from our point of view.’ Sherborn was of course a scientist and bibliographer, not an historian. He continued, ‘This collection is of infinite value to the British Museum, for hundreds of them refer to specimens actually in the various departments of Geology and Zoology … and will be a mine of information on general and bibliographic questions.’ (Norman 1944) In this of course, Sherborn is absolutely right. Owen’s papers are invaluable, in identifying and giving context to specimens in the collections.

In rescuing Owen’s papers, Sherborn is owed a huge debt of gratitude. It was an extraordinary achievement, on top of all his other work, to bring order to this chaos and turn the mountain of paper into the invaluable collection it is today. Of course there are regrets about some of his decisions in editing and selling, but thanks to him, an immensely important archive still survives. He too was its beneficiary. Owen’s manuscripts, Sherborn wrote, ‘have been of the greatest service to me during the years I have held them in answering queries as to the date of publications, the movements of men, and other matters in connection with my *Index Animalium*.’

He needed all the help he could get. Such was the explosion in scientific literature in the first half of the 19^th^ century, that while it had taken him 12 years to complete the first volume, 1758–1800, it was to take Sherborn another 21 years, until 1933, and 10 volumes to complete the Index just to 1850. That was still fifty years short of his original goal of completing the century. When Sherborn was asked if he would add 1850–1900, he replied that the museum could not expect to find another man 'with his knowledge and capacity content to work for an honorarium'. Furthermore, as the scientific literature since 1850 was so enormous, it would require a team of workers whose combined salaries would be something like £2000 a year – and that was simply unaffordable. He actually compiled a 'Time Sheet', which, he wrote, would 'be wanted should the Trustees continue the work after 1850. CDS'. It is neatly laid out and states that: 'It takes 1 month to edit 10,000 slips (c.3 feet?)... 1 month to look into queries of same...1 hour to number 1500 slips...18 years to amass the ms. 1801-1850...5 years to print A-L...' and so on. (Sherborn 1924)

**Figure 13.**
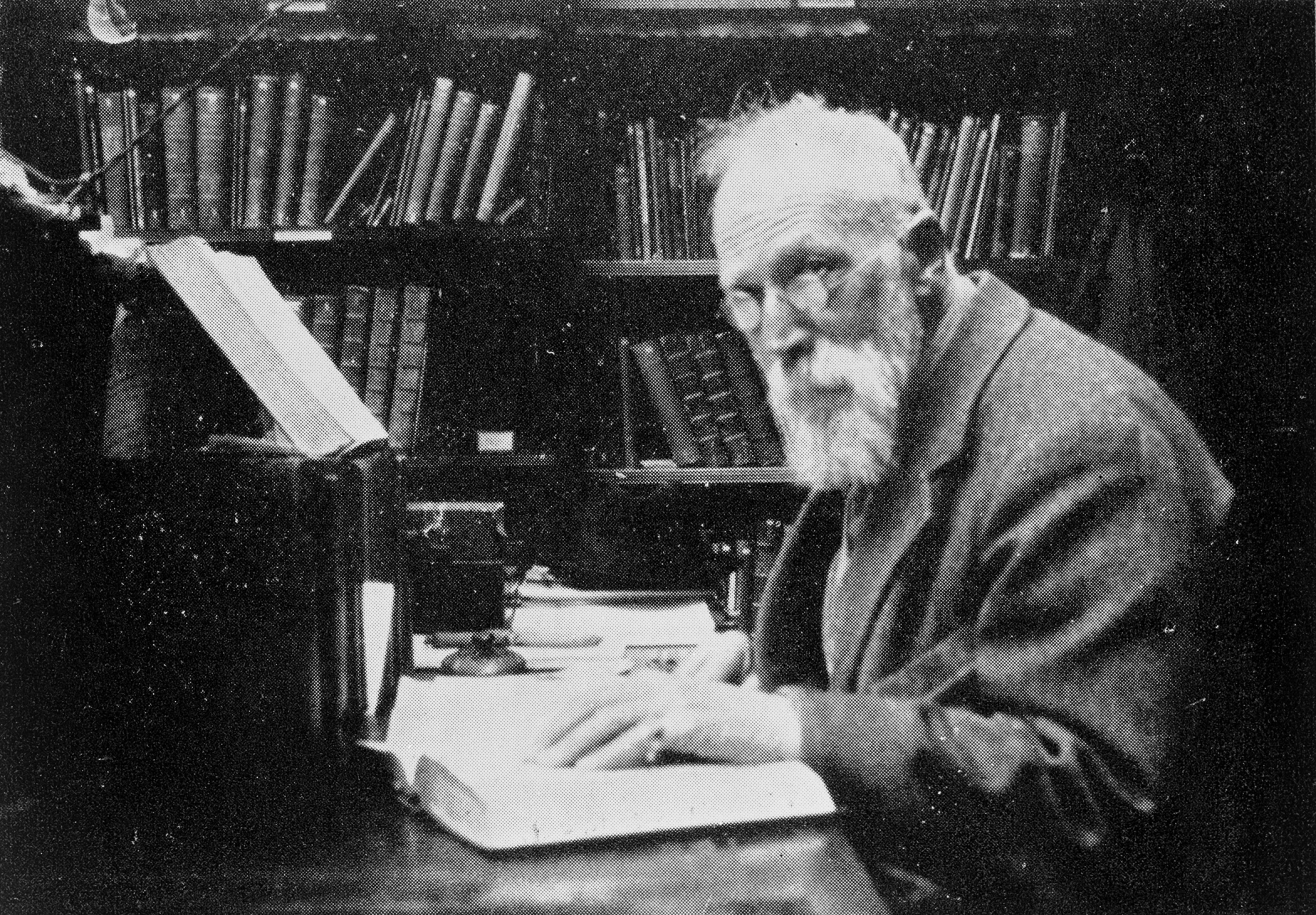
Sherborn at his desk in the Library of the NHM was the first port of call for generations of scientists seeking advice, information – or an invitation to one of his famous ‘smoke and chat’ parties. (With permission of The Trustees of the Natural History Museum, London)

The *Index* was received with the gratitude and admiration Sherborn so richly deserved. The compliments came from all over the world, but this, from Dr Bashford Dean of the American Museum of Natural History in 1923, sums up the general response. In a letter to the Director, he wrote: ‘I am very glad indeed to have this monumental work in my library, and I congratulate the South Kensington Museum [sic] with all my heart at completing the next stage of a magnum opus…such a work as Sherborn’s is a labor of love of the greatest magnitude, and I feel sure that the ‘index’ field covering the whole of the animal kingdom will be a boon to zoologists the world over for all time’. [Dean 1923]

Apart from 5000 entries made ‘by various friends abroad’, every entry, Sherborn wrote, ‘has been recorded from the original, arranged, sorted, checked, and passed for press by myself’. This was a true labour of love – for shamefully little financial reward. Sherborn compiled it single-handedly, and it took him 43 years, from 1890 to 1933, publishing each section as it was completed. His error rate was surprising low (Welter-Schultes et al. 2016) and the work formed the foundation for other similar projects with more focused taxonomic reach (e.g. Dickinson 2016). His reward was an honorary doctorate from Oxford, which gave him enormous pleasure – though to the end of his life he regretted that his mother had died before it was awarded to him. He also received the congratulations of the Trustees of the Natural History Museum, for which he had laboured for most of his life, though he never became a member of staff or received a regular salary or pension.

In addition to his work on the *Index*, he was president or fellow of various learned societies, wrote nearly 200 books and papers, including important contributions on microfossils (Miller 2016) and catalogues of natural history collections (Taylor 2016). He also catalogued the collections and library of the Geological Society of London. His interests were eclectic and wide-ranging and he was an avid collector of books, pictures and all kinds of antiquities. In a small notebook he would glue snippets of interesting stories or facts cut from newspapers, and jot down notes on pretty much anything that caught his eye. These ranged from the history of taxation, cures for the common cold, the derivation of symbols, and the Admiralty rules for the proportions of the Union Jack, to the best way to clean white marble – common washing soda and soap powder, or so he wrote. He was an enthusiastic theatre-goer, often seeing two or three productions a week. Almost inevitably, he collected theatre memorabilia, filling large volumes with reviews, posters, programmes and photographs of the theatrical stars of the age (Sherborn 1887–1942).

How he managed with so little income is extraordinary, but he was frugal in the extreme and cared little for his appearance. He felt the cold keenly, and would keep warm by inserting a sort of apron of felt beneath his clothes. In extremis, he would also use a newspaper. Instead of a necktie he wore a folded bit of red or black material drawn through an old gold ring. After his death, his friend and biographer John Roxborough Norman discovered that the black fabric had been cut from an old umbrella (Norman 1944).

**Figure 14.**
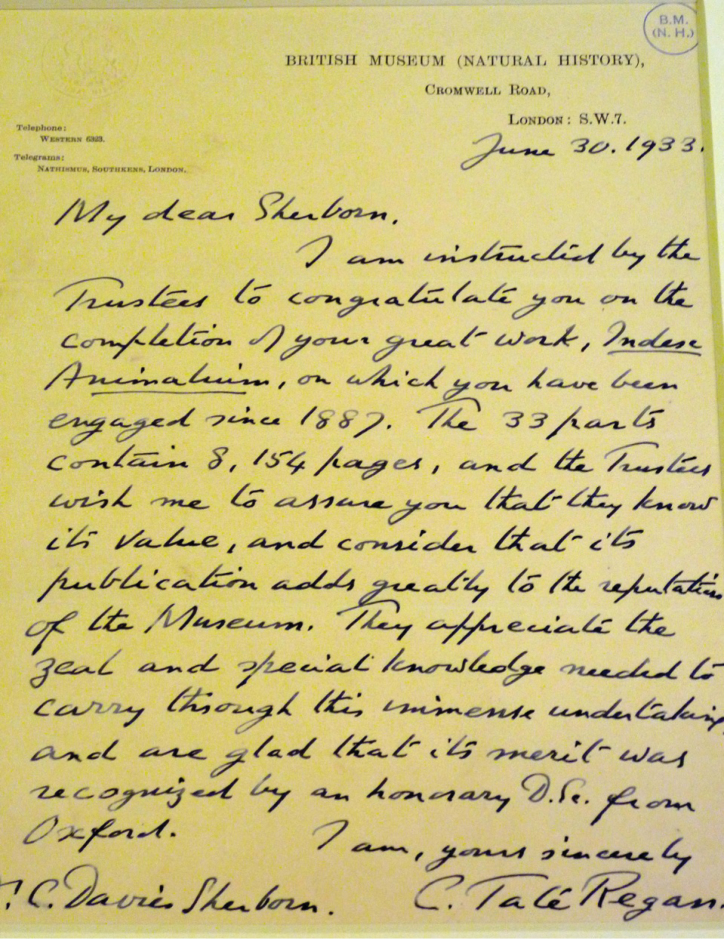
Letter from the Museum’s Director, Charles Tate Regan, on behalf of the Trustees, congratulating Sherborn on the completion of his ‘great work’ in 1933. Although he laboured for most of his life on behalf of the Museum, Sherborn never became a member of staff or received a regular salary or pension. (With permission of The Trustees of the Natural History Museum, London)

By the time of his death in 1942, Sherborn had become a vital pillar of the Natural History Museum. His corner in the museum library was the first port of call for generations of scientists – anyone who wanted to know anything went to Sherborn for information, advice, or to consult the ever-expanding boxes of *Index Animalium* slips, which from the outset provided an invaluable resource to the museum’s staff. (Harmer 1921). He was known by colleagues as ‘Squire’: JR Norman records that his family had links to the old manorial title of Squire of the Fawns and Cock Bell at Bedfont. To his junior colleagues he was Sherb, while to family and his closest friends he was Sherby or Charlie.

In the course of his work, Sherborn came across many volumes he thought should be in the Natural History Museum’s libraries. If he failed to persuade the museum to buy them, he bought them himself – and then either sold them to the museum for what he had paid for them, or simply donated them. One work, he has noted, was ‘Bought by CDS after refusal by Zool. Dept & presented 1930.’ Between 1891 and 1939 he acquired some 1600 volumes for the museum.

Sherborn’s generosity was not just to the museum’s libraries. His friends were beneficiaries of his great kindness and his collections. ‘No man was ever more generous’, recalled Francis Griffin of the Society for the Bibliography of Natural History (Sherborn was its first president), ‘in handing over treasures to his friends’. Griffin himself had received an armoured breastplate from Sherborn with the injunction to wear it under his jacket - ‘Much the easiest way of carrying it you know!’ (Griffin 1953)

As a young man new to the museum, Sherborn had been hugely appreciative of the ‘at homes’ given by Henry Woodward, Keeper of Geology. He carried that on with his own institution – his famous ‘smoke and chat’ parties. Sherborn believed strongly in the importance of bringing people together, and that it was the responsibility of senior colleagues to entertain their juniors and visitors in their homes. Today he would have been a sought-after networker. ‘A cup of coffee and a few biscuits all round’, was what he offered, together with ‘a good mix of chaps’. He invited not just scientific staff, but all grades in the museum, as well as foreign visitors and non-scientific friends.

These were informal, all male events. His biographer and friend, JR Norman quotes him as saying, ‘There’s no need to make it a social affair, with a lot of women and boiled shirts’, and with that attitude, it is perhaps not surprising that Sherborn acquired a reputation as a misogynist. Norman, however believed it may well have been a pose, but it was one Sherborn pursued with some vigour, even writing, ‘I never liked women and never chose them as companions’. He also deplored the ‘amazing standard of ignorance in the so-called educated woman’, complained they did not read the great masterpieces and ‘that is why the bulk of women are so extraordinarily uninteresting’.

Disparaging and sexist as these remarks are, the reality of his relationships with women seems very different. I knew from my research into the life of the palaeontologist Dorothea Bate who was associated with the museum for more than fifty years, how generous and kind he had been to her, and so he seemed to be generally with his female acquaintances.

**Figure 15.**
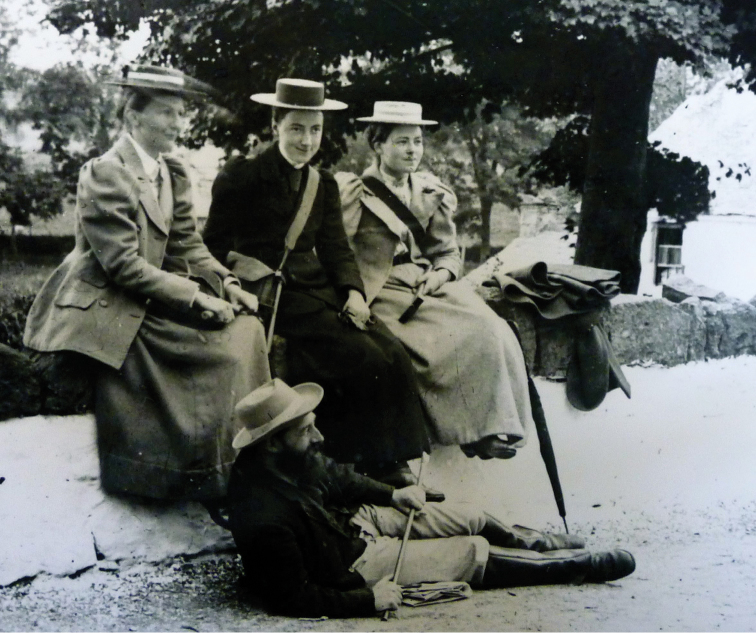
Sherborn at the feet of three unknown friends. (With permission of The Trustees of the Natural History Museum, London)

Indeed, women were among his closest friends and correspondents and, as he revealed to Norman, as a young man he had actually once contemplated marriage. ‘I once esteemed a woman very highly and engaged to marry her,’ he told Norman, ‘she was highly intelligent and well read’. However, his career came first, he realised he had no prospect of keeping her in comfort, and after ten years they parted, ‘and she wisely married another’. Whether his subsequent attitude was to disguise a profound hurt is impossible now to know, but Norman notes his views on marriage were largely cynical. When Norman himself married, Sherborn sent him this:

‘Dear JR,

Cheerio!

You have my entire sympathy.

Yours ever,

C. Davies Sherborn

Still raining!’ (Norman 1944)

One of his greatest friends and confidantes was Agnes Arber, to whom he wrote constantly. She also happened to be a botanist, philosopher and Fellow of the Royal Society. Sherborn professed to be uninterested in children, yet his papers reveal how witty and charming he was to the offspring of his numerous friends. To Mrs Arber’s daughter, Muriel, who had been unwell, he sent a sketch of himself in 1922 when she was nearly nine, and wrote:

‘Dear Muriel,

This is me. All you would see of me if you found me in the field. I am having a long rest in a few days.

I hope you are quite well again. It is no use being ill, it is such a waste of time.

Your friend,

C. Davies Sherborn

The sketch shows Sherborn, invisible apart from a large hat and puffs of smoke from his ubiquitous pipe, lying in grass under a tree with rabbits hopping happily around. (Sherborn 1922). Muriel, incidentally, was to become a respected geologist and teacher. (Robinson 2007).

The last years of Sherborn’s life were beset by ill-health, although he still went daily to the museum. A tremor in his hands made writing agonizingly difficult and he suffered increasingly from severe colds, though that did not persuade him to give up his beloved pipe. By 1940, as he wrote to a friend, the effect of the blitz meant he had ‘nothing to do at the Museum now, so stay put and read and smoke’. His visits to the Museum were reduced to Fridays only, and his housekeeper told Norman how lonely he was. In December 1941 he wrote, ‘Shall be alone Xmas Day, five sardines for dinner and a good pipe after’. His last visit to the museum was on Friday, 19 June 1942, when he met his friends afterwards at Lyons teashop in South Kensington as they had done for so many years. The following Monday as he was running a bath, he had a heart attack. He rallied a little when the doctor came, but he died that afternoon. Sherborn was cremated at Golders Green crematorium in north London the following Friday, at the exact time, Norman noted, that he would have been meeting his friends at Lyons teashop.

**Figure 16.**
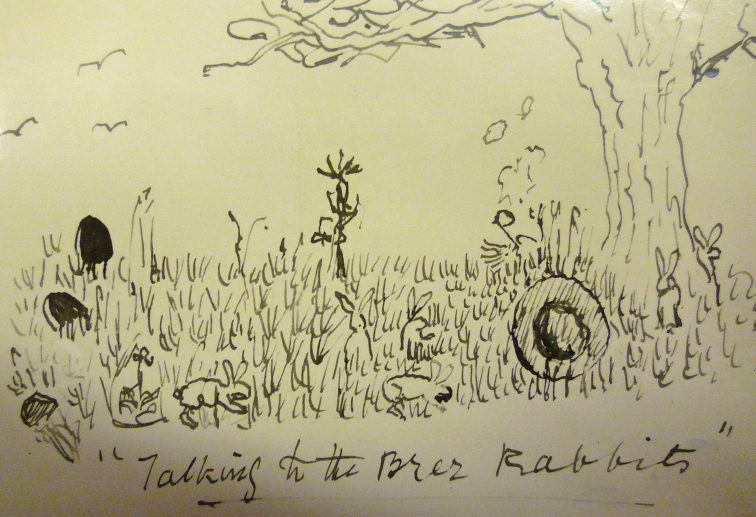
‘Talking to the Brer Rabbits’. Sherborn sent this drawing of himself in 1922 to the ‘nearly nine’ -year-old Muriel Arber who had been unwell. She was the daughter of his great friend, the botanist, philosopher and Fellow of the Royal Society, Mrs Agnes Arber. All that can be seen of Sherborn is a large hat and the smoke from his ubiquitous pipe. ‘I hope you are quite well again,’ he wrote to Muriel, ‘It is no use being ill, it is such a waste of time. Your friend, C. Davies Sherborn’. (With permission of The Trustees of the Natural History Museum, London)

Three months later, a letter arrived for him from an old friend, the Reverend CR Bower. Unaware that Sherborn had died, he wrote that he had been thinking a lot about him and had intended writing, but had been too busy. He asked his old friend to drop him a line: ‘we must not after all these years lose touch. Your friendship is one of my most treasured possessions. My wife,’ he ended, ‘sends her love’. (Bower 1942)

Charles Davies Sherborn’s contribution to zoology, bibliography and the collections of the Natural History Museum is unique. His rescue of Sir Richard Owen’s archive alone was of outstanding importance, but combined with his great memorial, the *Index Animalium*, this dedicated, idiosyncratic, self-taught man with an encyclopedic brain deserves to be celebrated and acclaimed with gratitude. Dr Dean of the AMNH could not have put it better in his letter to the NHM’s Director, Dr Sidney Harmer: ‘I think Sherborn is a marvel, and if he were not so devoted and patriotic, I tell you frankly that I would have stolen him bodily long since’.
